# Болезни гипофиза и COVID-19: систематический обзор литературы

**DOI:** 10.14341/probl13108

**Published:** 2022-07-20

**Authors:** У. В. Буйваленко, М. А. Перепелова, Р. А. Золотарева, Ж. Е. Белая, Г. А. Мельниченко

**Affiliations:** Национальный медицинский исследовательский центр эндокринологии; Национальный медицинский исследовательский центр эндокринологии; Национальный медицинский исследовательский центр эндокринологии; Национальный медицинский исследовательский центр эндокринологии; Национальный медицинский исследовательский центр эндокринологии

**Keywords:** COVID-19, коронавирус, SARS-CoV-1, SARS-CoV-2, гипофиз

## Abstract

С момента своего первого появления в декабре 2019 г. в Китае, в провинции Ухань, вирус тяжелого острого респираторного синдрома коронавируса 2 (SARS-CoV-2) быстро распространился по всему миру, вызвав беспрецедентную пандемию. На момент написания статьи заболевание перенесли более 400 млн человек, умерли почти 6 млн жителей Земли.

SARS-CoV-2, вызывающий COVID-19, получает клеточный доступ через рецептор ангиотензинпревращающего фермента 2 (AПФ2), который связан с трансмембранным белком сериновой протеазы 2 (TMPRSS2). У людей мРНК AПФ2 экспрессируется в нескольких эндокринных железах, включая гипофиз, поджелудочную железу, щитовидную железу, яичники и яички. Важно отметить, что мРНК TMPRSS2 также экспрессируется в гипофизе, поджелудочной железе, щитовидной железе, яичниках и яичках. Таким образом, эндокринная система обладает не только рецептором AПФ2, но и белком TMPRSS2, необходимым для доступа вириона SARS-CoV-2 к клеткам, что делает эндокринную систему особенно уязвимой как для разрушения, так и для изменения функции из-за COVID-19. Уже первые месяцы жизни с COVID-19 показали, что основными факторами риска тяжелого течения являются сахарный диабет, ожирение, гипертоническая болезнь, и фатальные исходы существенно чаще регистрируют у лиц, имеющих переломы тел позвонков. Важно заметить, что подобное сочетание патологических состояний закономерно выявляется у лиц с эндогенным и экзогенным гиперкортицизмом, которые также сопровождаются иммунодефицитными состояниями.

В настоящем обзоре мы представляем современные сведения о течении COVID-19 у пациентов с гипоталамо-гипофизарными заболеваниями, а также обсуждаем варианты лечения эндокринопатий при COVID-19.

## Abstract (en)

A severe acute respiratory syndrome coronavirus 2 (SARS-CoV-2) has rapidly spread around the world since was first scientifically described in December 2019. At present approximately 400 million people have suffered from the disease, almost 6 million people have died.

SARS-CoV-2 uses the angiotensin-converting enzyme 2 (ACE2) and the serine protease TMPRSS2 for S protein priming. ACE2 and TMPRSS2 are expressed in several endocrine glands, including the pituitary, pancreas, thyroid, ovaries, and testes. Thus, the endocrine glands may be a direct target for SARS-CoV-2. The main risk factors for severity of the COVID-19 are obesity, arterial hypertension, diabetes mellitus (DM), vertebral fractures, which potentially predisposes patients to a severe course of COVID-19.

In this review, we present current data on the course of COVID-19 in patients with hypothalamic-pituitary diseases, and also discuss treatment for endocrinopathies during to COVID-19.

## МЕТОДОЛОГИЯ ПОИСКА ИСТОЧНИКОВ

Обзор литературы проводился с использованием ключевых слов COVID-19, Coronavirus, SARS-CoV-1, SARS-CoV-2, Pituitary, Endocrine в базах данных: PubMed, Google Scholar и MedARXiv. Дополнительные ссылки выявлены путем ручного просмотра библиографий и цитирования в выбранных статьях. Были включены статьи, написанные на английском и русском языках. Последний поиск литературы осуществлялся 13 февраля 2022 г. По прошествии 2 лет в результате поиска с использованием данных ключевых слов в базе данных PubMed можно найти более 140 статей.

## ВВЕДЕНИЕ

В конце 2019 г. в Китайской Народной Республике (КНР) произошла вспышка новой коронавирусной инфекции, вызванной новым коронавирусом (SARS-CoV-2). 11 февраля 2020 г. Всемирная организация здравоохранения (ВОЗ) определила официальное название инфекции, вызванной новым коронавирусом, — COVID-19 [1]. Согласно секвенированию генома, SARS-CoV-2 является бета-коронавирусом того же подрода, что и SARS-CoV, с которым он имеет примерно 80% идентичных последовательностей [2]. Судя по сходству последовательностей РНК, вполне вероятно, что данные предыдущих сообщений о пациентах с инфекцией SARS можно частично экстраполировать на пациентов с SARS-CoV-2. Известно, что SARS-CoV-2 проникает в клетку через рецептор ангиотензинпревращающего фермента 2 (AПФ2), аналогично SARS-CoV [2]. Мишенями SARS-CoV-2 являются дыхательные пути и многие другие органы: желудочно-кишечный тракт, печень, почки, кожа, эндокринные железы, опорно-двигательный аппарат, центральная нервная и сердечно-сосудистая системы. Эпидемиологические данные первых случаев госпитализации пациентов с COVID-19 показали, что COVID-19 чаще поражает пожилых мужчин с хроническими сопутствующими заболеваниями, в основном метаболическими, сердечно-сосудистыми и цереброваскулярными [3].

Факторами риска развития острого респираторного дистресс-синдрома (ОРДС) и летального исхода являются ожирение, сахарный диабет, гипертоническая болезнь, сердечно-сосудистые и почечные заболевания [4].

Для COVID-19 характерно наличие клинических симптомов ОРВИ, однако в некоторых случаях симптомы могут отсутствовать [5][6].

Гипоталамо-гипофизарная ось (ГГО) играет важную роль в воспалительных или стрессовых реакциях при вирусных инфекциях. Воздействие на функцию ГГО может быть результатом прямого или косвенного повреждения. Прямое повреждение может быть связано с опосредованной рецептором АПФ2 инфекцией нервных клеток SARS-CoV-2, вызывающей отек и некроз клеток [7]. Непрямое воздействие опосредовано цитокинами: интерлейкины (ИЛ), ИЛ-1, -6, и фактор некроза опухоли-альфа (ФНО-α) активируют гипоталамо-гипофизарно-надпочечниковую ось (ГГНО) и впоследствии повышают уровень кортизола в сыворотке [8]. Также воспаление может привести к поражению гипофиза и гипоталамуса, вызывая гипофизит при SARS-CoV-2 [9].

Несмотря на изученные патофизиологические механизмы взаимодействия организма человека и вируса SARS-CoV-2, недостаточно данных для создания клинических рекомендаций для ведения пациентов с нейроэндокринными заболеваниями. Необходимо на клинических примерах оценить возможность комбинации противовирусной терапии с медикаментозным лечением по поводу основного заболевания, выявить показания/противопоказания при выборе хирургической тактики лечения, определить тактику ведения пациентов в послеоперационном периоде и пр., чему и посвящен настоящий систематический обзор литературы.

## ГИПОПИТУИТАРИЗМ

Гипопитуитаризм (гипоталамо-гипофизарная недостаточность) — заболевание, обусловленное отсутствием или снижением секреции гипоталамических нейрогормонов, а также одного или более гипофизарных гормонов. У пациентов с гипопитуитаризмом могут наблюдаться вторичный гипокортицизм, вторичный гипогонадизм, вторичный гипотиреоз, дефицит гормона роста, ожирение, несахарный диабет, что влияет на тяжесть течения новой коронавирусной инфекции. Интересно, что COVID-19 также может являться одной из причин возникновения гипопитуитаризма, провоцируя развитие апоплексии гипофиза — редкого синдрома, характеризующегося кровоизлиянием, чаще в опухоль гипофиза, признаками резко нарастающей внутричерепной гипертензии, нарушением зрения. Кроме того, гипофизиты могут быть характерным осложнением вирусной инфекции.

Alzahrani A. и соавт. сообщили о нарушении адренокортикального ответа при заболевании COVID-19. У 28 пациентов в утренние часы измеряли уровни кортизола, адренокортикотропного гормона (АКТГ) и дегидроэпиандростерона сульфата в плазме при поступлении в стационар и в течение госпитализации. Было выявлено нарушение адренокортикального ответа у значительного числа пациентов, уровни кортизола и АКТГ в плазме соответствовали центральной надпочечниковой недостаточности, что предполагает прямую связь между COVID-19 и нарушением функции ГГНО [10].

За время пандемии накопилось немало клинических описаний гипопитуитаризма в сочетании с COVID-19. Solorio-Pineda S. и соавт. сообщили о пациенте 27 лет с прогрессирующим снижением остроты зрения, связанным с симптомами респираторного заболевания и интенсивной головной болью. На КТ органов грудной клетки выявлен многодолевой инфильтрат с ретикулонодулярным рисунком, на КТ головного мозга — апоплексия в макроаденому гипофиза. Был подтвержден SARS-CoV-2 и начата респираторная поддержка, несмотря на которую пациент скончался вследствие легочных осложнений. Авторы задаются вопросом, была ли инфекция COVID-19 причиной гипофизарной апоплексии или это случайное событие. Однако очевидно, что вовлечение ЦНС при COVID-19 включает цереброваскулярные события из-за эндотелиальной дисфункции, при этом апоплексия гипофиза является необычным проявлением [11].

У другой больной, 74 лет, с выраженной головной болью, легкими симптомами респираторной инфекции, лабораторными данными гипофункции гипофиза при визуализации головного мозга выявлены МРТ-признаки аденомы гипофиза скровоизлиянием. В течение 24 ч после обращения развилось острое ухудшение зрения, повторная визуализация показала развивающееся кровоизлияние и сдавление перекреста зрительных нервов. Пациентке успешно провели экстренную эндоскопическую эндоназальную транссфеноидальную аденомэктомию, несмотря на ее положительный тест на COVID-19, с принятием специальных интраоперационных мер предосторожности для снижения риска передачи SARS-CoV-2. В послеоперационном материале выявлены изменения, соответствующие апоплексической аденоме гипофиза, с пролиферативным индексом Ki-67 примерно от 1 до 2%. По результатам иммуногистохимического анализа (ИГХ) — положительное окрашивание на фолликулостимулирующий гормон, синаптофизин и GATA3 в жизнеспособных клетках. В послеоперационном периоде развились вторичная надпочечниковая недостаточность и гипотиреоз, вскоре пациентка выписана с улучшением на заместительной терапии. Авторы предположили, что SARS-CoV-2 может влиять на каскад свертывания крови, вызывая тромбоцитопению и дисфункцию тромбоцитов, а в сочетании с гиперстимуляцией гипофиза в условиях острой инфекции это потенциально может спровоцировать инфаркт гипофиза и/или кровоизлияние [12].

Kamel W. и соавт. сообщили о мужчине 55 лет с подтвержденной инфекцией COVID-19, поступившем с прогрессирующим снижением остроты зрения и параличом глазодвигательного нерва. В анамнезе: сахарный диабет, гипертония, резекция макроаденомы гипофиза 11 лет назад. Пациент находился на заместительной терапии по поводу пангипопитуитаризма. По данным МРТ при поступлении — признаки апоплексии макроаденомы гипофиза. Проведена повторная частичная эндоназальная транссфеноидальная аденомэктомия. В послеоперационном периоде отмечено незначительное улучшение зрения и глазодвигательного паралича. При ИГХ-исследовании выявлены классические признаки апоплексии гипофиза. Однако, несмотря на проведенное оперативное лечение, дыхательная недостаточность прогрессировала, пациент был переведен в отделение интенсивной терапии, где начата искусственная вентиляция легких (ИВЛ). Через неделю пациент скончался из-заосложнений тяжелой пневмонии, вызванной COVID-19 [13].

Описана апоплексия гипофиза (при ранее не диагностированной аденоме) на фоне СOVID-19 в III триместре беременности. При этом при поступлении в стационар пациентку в течение 4 дней беспокоили нечеткое зрение, расширенный левый зрачок и головная боль, был выявлен положительный тест на SARS-СoV-2. Пациентке назначили дексаметазон по 4 мг 2 раза в день, и в течение 12 ч левый зрачок уменьшился в размере до 4 мм и стал более реактивным на свет, без каких-либо других изменений при осмотре. Недавние рекомендации Fleseriu M. и соавт. предлагают классифицировать пациентов с положительным результатом на COVID-19, нуждающихся в хирургическом лечении, как экстренных, неотложных или плановых взависимости от сроков оперативного вмешательства [14]. Принимая во внимание стабилизацию симптомов на фоне консервативной терапии, методом выбора лечения стало плановое оперативное вмешательство после родоразрешения. Во время проведения трансназальной транссфеноидальной аденомэктомии идентификация гипофиза оказалась невозможной, определялась геморрагическая масса с фибрином. Через 2 мес после оперативного лечения развились вторичный гипотиреоз и вторичный гипогонадизм [15].

Таким образом, сравнительный анализ вышеописанных 4 случаев не выявил корреляции тяжести течения СOVID-19 с возрастом и полом пациентов с нейроэндокринными заболеваниями, однако наличие пневмонии свидетельствует о более высоком риске тяжелого течения СOVID-19. Также очевидно, что проведение трансназальной аденомэктомии возможно при наличии соответствующих показаний и соблюдении специальных мер безопасности (табл. 1).

**Table:** Таблица 1. Сравнительный анализ случаев пациентов с гипопитуитаризмом и СOVID-19Table 1. Comparative analysis of cases of patients with hypopituitarism and COVID-19

Пациент	1	2	3	4
Возраст	27	74	55	28
Пол	м	ж	м	ж
Наличие пневмонии	+	-	+	-
Положительный ПЦР-тест на COVID-19	+	+	+	+
Головная боль	+	+	+	+
Ухудшение зрения	+	+	+	+
Паралич глазодвигательного нерва	-	+	+	-
Анизокория	+	-	-	+
Проведенная трансназальная аденомэктомия	-	+	+	+
Исход	Смерть	Выздоровление	Смерть	Выздоровление

## ГИПЕРКОРТИЦИЗМ И COVID-19

У здоровых людей в ранней фазе инфекционного процесса глюкокортикоиды (ГК) оказывают иммуностимулирующее действие, повышая чувствительность иммунной системы к внешним агентам [16]. Помимо известного противовоспалительного и противоаллергического действия, имеются данные об усилении местной врожденной иммунной защиты в клетках респираторного эпителия в ответ на воздействие ГК [17]. Однако со временем и при стабильно высоких концентрациях основные эффекты ГК заключаются в глубокой иммуносупрессии с подавлением врожденного и адаптивного иммунного ответа. Таким образом, хронический избыток ГК препятствует активации адаптивных реакций у пациентов с гиперкортицизмом. Впоследствии снижение числа В-лимфоцитов и Т-лимфоцитов, а также активации Т-хелперов способствует развитию оппортунистической и внутриклеточной инфекций, а сочетание генерализованной иммуносупрессии с нарушением иммунного ответа — хроническим вялотекущим воспалительным процессам, что является причиной большого количества клинических осложнений [18]. Известно, что ранее существовавшие хронические воспалительные заболевания повышают риск смерти, связанной сCOVID-19 [19]. Помимо метаболических изменений, у пациентов как с эндогенным, так и с ятрогенным гиперкортицизмом повышен риск различных бактериальных, вирусных, грибковых и паразитарных инфекций, вызванных иммунодефицитом [20–22].

Эндогенный гиперкортицизм, то есть эндогенный синдром Кушинга, является редким заболеванием с распространенностью 1,2–2,4 на 100 000 населения [23], тогда как супрессивные дозы ГК получают 1–2% населения, и ятрогенный гиперкортицизм развивается примерно у 70% из них [24][25]. По данным литературы, прием ГК, а также эндогенный избыток позволяют смягчить гипериммунные реакции, ответственные за тяжелое прогрессирование заболевания COVID-19 [26].

В исследовании RECOVERY установлено, что введение дексаметазона 6 мг ежедневно в течение 10 дней является эффективной терапией пациентов, нуждающихся в высокопоточном кислороде или ИВЛ, однако ухудшает прогноз у больных с легким течением COVID-19 [27][28]. Вместе с тем применение экзогенных ГК, как и заболевания, сопровождающиеся избытком эндогенной продукции ГК, часто ассоциированы с такими побочными эффектами, как ожирение, артериальная гипертензия, сердечно-сосудистые заболевания, стеатоз печени, инсулинорезистентность и сахарный диабет 2 типа [24, 29]. Интересно, что более ранние наблюдения по применению стероидов при ближневосточном респираторном синдроме (MERS) и SARS [30] давали основание скептически относиться к их использованию.

ГК надпочечников реализуют множество эффектов в организме человека: противовоспалительное действие проявляется в виде антиэкссудативных и антипролиферативных эффектов, ГК влияют на обменные процессы, обладают противоаллергическим и иммунодепрессивным действиями. Регуляция выделения ГК контролируется гипоталамо-гипофизарным механизмом. При уменьшении концентрации свободного кортизола гипоталамус выделяет кортикотропин-рилизинг-гормон, который стимулирует высвобождение АКТГ, который, в свою очередь, обуславливает выброс ГК корой надпочечников [31]. Существует гипотеза о том, что эндогенный кортизол действует через активацию незащищенных минералокортикоидных рецепторов и через этот механизм проявляет негативные эффекты при заболевании COVID-19 [32][33]. Поскольку дексаметазон, в отличие от кортизола, более избирательно связывается с ГК-рецептором, он подавляет секрецию эндогенного кортизолаи проявляет лишь слабое минералокортикоидное действие. Возможно, подобное предположение объясняет позитивный эффект дексаметазона у пациентов с COVID-19 в тяжелом состоянии.

Высказано предположение, что резистентность к ГК способствует увеличению заболеваемости и смертности при COVID-19 за счет уменьшения способности ингибировать воспаление, вызванное SARS-CoV-2 [34], или посредством активации минералокортикоидных рецепторов, как это установлено при семейных синдромах резистентности к глюкокортикоидам [35].

Первые рекомендации по тактике ведения эндокринопатий в пандемию появились уже в 2020 г. и затрагивали вопросы, касающиеся больных с гиперкортицизмом [14][36][37]. Подчеркивалось, что такие пациенты относятся к группе высокого риска тяжелого течения COVID-19. Однако данные о влиянии избытка ГК на течение ассоциированных с COVID-19 состояний во многом противоречивы, поэтому особенности течения заболевания у лиц с природной «моделью» хронического избытка кортизола представляются чрезвычайно важной информацией.

Медицинским сообществом были предложены превентивные меры: телемедицинские консультации и соблюдение социальной дистанции, рекомендации по заочной коррекции медикаментозной терапии в тех случаях, когда не выявлено необходимости в ургентном хирургическом вмешательстве [38]. Несмотря на то что оперативное лечение является методом выбора, во время пандемии может быть целесообразна отсрочка операции [14] как с позиций безопасности больного, так и спозиций защиты медицинского персонала. Оперативные вмешательства на аденогипофизе с трансназальным доступом в пандемию могут выполняться только по ургентным показаниям.

Для коррекции гиперкортицизма рекомендована кортизолснижающая терапия ингибиторами стероидогенеза [14][39]. Из-за быстрого начала действия и эффективности предпочтение следует отдавать кетоконазолу, метирапону и осилодростату [40][41]. К моменту распространения COVID-19 в РФ был доступен только кетоконазол (off label), метирапон в настоящее время не применяется, а осилодростат могли получать только те пациенты, которые принимали участие в клинических исследованиях, уже завершившихся к началу пандемии. Митотан, имеющийся в РФ, позиционируется как препарат для лечения адренокортикального рака, а мифепристон может быть использован только off-label и, поскольку его побочным эффектом является повышение артериального давления, — с особой осторожностью. Недостатками кетоконазола являются не только его достаточно частые нежелательные влияния на печень, но и способность увеличивать интервал Q–T, кроме того, препарат интерферирует со многими лекарственными молекулами, влияя на CYP3A4.

В случае развития COVID-19 у пациентов с предшествующей кортизолснижающей терапией следует назначать стрессовые дозы ГК для предотвращения надпочечниковой недостаточности [39]. Иными словами, тактика ведения пациентов не отличается от той, которую рекомендуют при гипокортицизме, т.к. неизвестно, смогут ли надпочечники к моменту инфицирования физиологически отреагировать на инфекцию подъемом продукции ГК. В определенных ситуациях схемы «блокируй и замещай» также могут быть полезны [14][39]. У пациентов с высокими дозами экзогенных ГК рекомендуется минимально возможная дозировка препаратов, у пациентов с эндогенным гиперкортицизмом — адекватный контроль основного заболевания. Кроме того, мониторинг сопутствующих метаболических и сердечно-сосудистых заболеваний является особенно важным для улучшения профиля риска у пациентов с избытком ГК.

В настоящее время для пациентов с гиперкортицизмом и COVID-19, поскольку оба состояния связаны с высоким риском тромбоэмболии [40–42], рекомендуется коррекция гиперкоагуляции, которая вызывает капиллярный, венозный и/или артериальный тромбоз [43][44]. Наряду с гиперкоагуляцией пациенты с гиперкортицизмом и COVID-19 подвержены высокому риску вторичных бактериальных или грибковых инфекций [45], поэтому необходима эмпирическая профилактика антибиотиками широкого спектра действия, особенно у госпитализированных пациентов [18].

Опасения относительно возможного повышенного риска неблагоприятных исходов у пациентов с гиперкортицизмом, принимающих ингибиторы АПФ или блокаторы рецепторов ангиотензина, которые могут повышать уровень экспрессии рецепторов АПФ2, давно утратили актуальность [46]. Не рекомендуется прекращать прием этих препаратов из-за отсутствия достаточных доказательств, а также потому, что это может усугубить сердечно-сосудистые заболевания и болезни почек [47][48]. Медикаментозная терапия сопутствующих заболеваний остается без изменений, при признаках декомпенсации сахарного диабета необходим перевод с пероральных сахароснижающих препаратов на инсулин. При тяжелом состоянии прием пероральных бисфосфонатов, которые ранее использовал больной, необходимо прекратить. Точных данных о клиническом исходе COVID-19 от терапии деносумабом еще не получено, однако, вероятнее всего, ее не стоит откладывать более чем на 1 мес [49].

Связаны ли положительные эффекты дексаметазона при лечении COVID-19 с иммуностимулирующим эффектом ГК у пациентов в критическом состоянии, «смягчением» синдрома высвобождения цитокинов и ОРДС, лечением недиагностированной надпочечниковой недостаточности или подавлением секреции эндогенного кортизола со сниженным влиянием на минералокортикоидные рецепторы, предстоит уточнить в будущем.

В первые дни работы в условиях новой коронавирусной инфекции в ФГБУ «НМИЦ эндокринологии» МЗ РФ были получены данные о трех пациентах с активной болезнью Иценко–Кушинга [50]. Первая пациентка, женщина, 71 год, поступила за неделю до перепрофилирования центра в инфекционную больницу для работы с больными COVID-19 и прекращения плановой помощи. В связи с остро развившимися опасными для жизни осложнениями (гипокалиемия, анемия, гипоальбуминемия, гипергликемия (17 ммоль/л), почечная недостаточность (скорость клубочковой фильтрации — 22 мл/мин/1,73 м2)) пациентка госпитализирована в отделение интенсивной терапии и реанимации. Пожилой возраст, тяжелые метаболические нарушения, особенности анамнеза (20-летний стаж сахарного диабета 2 типа и отсутствие, по данным медицинской документации, при амбулаторном обследовании 3 мес назад признаков эндогенного гиперкортицизма) свидетельствовали о крайне быстром развитии гиперкортизолизма и высокой вероятности синдрома эктопированной продукции АКТГ. Учитывая тяжелое течение заболевания, для экстренной двусторонней адреналэктомии переведена в другую больницу на фоне коррекции гипокалиемии и метаболических нарушений. Через 6 дней состояние пациентки ухудшилось, появилась одышка, получен положительный результат теста на SARS-CoV-2. КТ грудной клетки показала двусторонние диффузные изменения по типу матового стекла (GGO)и двусторонний плевральный и перикардиальный выпот. В соответствии с критериями cHIS (COVID-19-associated hyperinflammatory syndrome) отмечалась гематологическая дисфункция при типичных признаках гипервоспалительного синдрома, ассоциированного с COVID-19 [51][52]. Разумеется, проведение адреналэктомии в таких условиях, даже по ургентным показаниям, было невозможным, поскольку многократно увеличивался риск инфицирования и тромбозов. Респираторный статус пациентки в течение следующих 4 дней резко ухудшился с прогрессированием и развитием ОРДС, требовавшего проведения ИВЛ, на КТ через 3 дня выявлено поражение 80% легочной ткани с последующим наступлением летального исхода. Официально подтвержденная причина смерти — COVID-19, осложнившийся двусторонней полисегментарной геморрагической пневмонией и ОРДС с сопутствующим заболеванием «эндогенный гиперкортицизм». В патологоанатомическом заключении не было упоминания об опухоли, продуцирующей АКТГ, что могло быть связано с небольшим размером опухоли или недоступностью посмертной дифференциальной диагностики, не позволившей провести достаточно тщательное обследование пациентов с COVID-19. Таким образом, развитие COVID-19 у больной с нелеченым тяжелейшим гиперкортизолизмом полностью соответствовало представлениям о потенциально фатальном риске COVID-19 у подобных больных.

Вторая женщина, 38 лет, находилась под наблюдением в ФГБУ «НМИЦ эндокринологии» с длительным (5 лет) анамнезом болезни Иценко–Кушинга, перенесла две транссфеноидальные аденомэктомии в период с 2015 по 2020 г., по результатам ИГХ-исследования подтверждено наличие АКТГ-продуцирующей опухоли гипофиза с Ki-67 9,11% в 2015 г. и с Ki-67 30,2% в 2020 г. В послеоперационный период отмечались улучшение самочувствия, нормализация лабораторных показателей, однако затем наступала активная стадия болезни Иценко–Кушинга. В течение 5 лет наблюдения больной неоднократно назначали пасиреотид и кетоконазол, от приема которых она отказывалась из-за плохой переносимости. Было рекомендовано радиохирургическое лечение, однако оно было отложено из-за начала пандемии. В апреле 2020 г. пациентка госпитализирована с положительным тестом на SARS-CoV-2, одышкой, кашлем, болью в груди и лихорадкой с максимальным повышением температуры тела до 39,3 °С. На КТ органов грудной клетки — двустороннее поражение легких. Инициированы оксигенотерапия, антибиотикотерапия и симптоматическое лечение парацетамолом, дексаметазон не назначали. Значительное клиническое улучшение достигнуто на 14-й день госпитализации. На 24-й день пациентка была выписана из больницы с улучшением после двойного отрицательного результата теста на SARS-CoV-2. Три недели спустя при КТ были выявлены ранние признаки фиброза легких.

Третья пациентка, 66 лет, наблюдалась в ФГБУ «НМИЦ эндокринологии» по поводу болезни Иценко–Кушинга около 4 лет. Диагноз был поставлен на основании селективного забора крови из нижних каменистых синусов, а также по данным ИГХ-исследования после нейрохирургического лечения кортикотропиномы. В послеоперационном периоде достигнута лабораторная ремиссия, однако в дальнейшем возникали рецидивы. Был рекомендован прием пасиреотида, но пациентка от терапии отказалась. Во время планового скрининга в мае 2020 г. у пациентки обнаружен положительный результат на SARS-CoV-2. На КТ органов грудной клетки выявлены типичные признаки пневмонии с поражением легких 4%. С целью профилактики возможных осложнений пациентка была госпитализирована. На осмотре при поступлении признаков патологии дыхательной и сердечно-сосудистой систем не выявлено. Симптомов кашля, лихорадки (t=36,5°C) или затрудненного дыхания не было. Поданным лабораторного обследования была незначительно повышена скорость оседания эритроцитов, остальные результаты находились в пределах нормы. При оценке активности основного заболевания лабораторные показатели оказались в пределах референсного диапазона, кроме ночного уровня кортизола сыворотки крови, который был повышен. Кроме инсулинов и антигипертензивных препаратов, пациентка больше ничего не получала. Бессимптомная форма COVID-19 с поражением легких 4% через 10 дней лечения завершилась полным выздоровлением.

Поскольку особенно тяжелым течением отличалась пациентка с впервые диагностированной болезнью Иценко–Кушинга, авторы предположили, что клиническое течение COVID-19 зависит от тяжести гиперкортицизма. Помимо этих случаев из нашего центра, к настоящему времени известно еще о нескольких клинических наблюдениях развития COVID-19 у больных с гиперкортицизмом.

Yuno A. и соавт. сообщили, что из 61 больного с болезнью Кушинга, находящихся под их наблюдением, инфицирование SARS-СoV-2 было выявлено у 3,2% больных [44]. Авторы также описали случай успешного лечения пациентки с активной формой болезни Кушинга в сочетании с COVID-19. Женщина, 27 лет, медицинский работник, у которой было запланировано нейрохирургическое лечение по поводу болезни Кушинга, после тесного контакта с пациентом с COVID-19 отметила появление одышки, при КТ органов грудной клетки диагностирована вирусная пневмония. Несмотря на отсутствие известных факторов риска, связанных с тяжестью течения COVID-19, респираторный статус пациентки ухудшился, возникла потребность в оксигенотерапии (со скоростью 7 л/мин через маску). Инициировано медикаментозное лечение по схеме «блокируй и замещай» с использованием ингибиторов стероидогенеза и гидрокортизона для контроля гиперкортицизма, для лечения пневмонии использовались противовирусные препараты, а также симптоматическая терапия. Через 1 мес после отрицательного результата теста на SARS-CoV-2 была выполнена аденомэктомия. В послеоперационном периоде достигнута лабораторная ремиссия болезни Кушинга. Авторы пришли к выводу, что гиперкортицизм вследствие активной формы болезни Кушинга может усугубить течение инфекции COVID-19.

Веretta F. и соавт. описали начало развития надпочечниковой недостаточности у пациента 67 лет с болезнью Кушинга из-за рецидива агрессивной кортикотропиномы на фоне лечения метопироном и каберголином при заболевании COVID-19 [53].Пациент госпитализирован в отделение с вирусной пневмонией в критическом состоянии. Из анамнеза известно, что дважды оперирован по поводу АКТГ- продуцирующей макроаденомы, проведена лучевая терапия, получал заместительную терапию (левотироксин, кортизона ацетат). В 2013 г. случился рецидив гиперкортицизма, кортизона ацетат отменен, инициирована терапия пасиреотидом и каберголином. Далее отмечен повторный рост аденомы гипофиза, в 2017 г. снова прооперирован по поводу компрессии правой орбиты (при ИГХ-исследовании — ткани с АКТГ-продукцией). В мае 2018 г. к терапии добавлен метирапон. В 2018 г. получал химиотерапию по поводу прогрессирующего увеличения размера опухоли. В феврале 2020 г. были обнаружены признаки тромбоза глубоких вен на правом плече, инициирована терапия эноксапарином 8000 МЕ подкожно 2 раза в день. На момент поступления коррекции требовали надпочечниковая недостаточность и персистирующая гиперкалиемия. Случай закончился выздоровлением, однако самое интересное заключается в выводах. Авторы раскрывают возможность титрования или временной приостановки медикаментозной терапии эндогенного гиперкортицизма в контексте инфекции COVID-19и подчеркивают, что персистирующая гиперкалиемия у пациентов с синдромом Кушинга возникает при лечении низкомолекулярными гепаринами и может быть сигналом подавления продукции альдостерона.

Уникальное наблюдение представлено de Filette J. и соавт. [53]. Мужчина, 43 года, с АКТГ-секретирующей карциномой с метастазами в мозжечок и тяжелым течением COVID-19, ранее оперированный на гипофизе, перенесший радиотерапию, получавший кетоконазол, пасиреотид, каберголин, подвергшийся субтотальной билатеральной адреналэктомии и химиотерапии темозоломидом, к которой была добавлена комбинированная терапия чек-пойнт ингибиторами — ипилимумабом иниволумабом. Течение болезни было стабильным, планировавшаяся резекция остаточной ткани левого надпочечника была отложена из-за присоединившегося COVID-19. В марте 2021 г. в отделении ОРИТ сатурация кислорода в крови — 72%, инициирована высокопоточная оксигенация, в дальнейшем — ИВЛ. Введение дексаметазона начато с момента поступления, использованы антибиотики широкого спектра и низкомолекулярный гепарин. Схема «блокируй — замещай» с использованием кетоконазола и гидрокортизона начата с 11-го дня, после отмены дексаметазона. Несмотря на полиорганное поражение, включая метаболический ацидоз и острую почечную недостаточность, острый коронарный синдром 2 типа, септический тромбофлебит и полинейропатию, больной в настоящее время, после неоднократных поступлений в реанимацию, находится на домашней реабилитации.

Таким образом, хронический избыток ГК повышает риск смерти, связанной с COVID-19, вместе с тем, помимо метаболических изменений, у пациентов как с эндогенным, так и с ятрогенным гиперкортицизмом повышен риск различных инфекций, вызванных иммунодефицитом, что диктует необходимость проведения международных многоцентровых исследований и дальнейшего изучения влияния COVID-19 на течение гиперкортицизма.

## АКРОМЕГАЛИЯ

Акромегалия — это редкое заболевание с распространенностью 2,8–13,7 на 100 000 населения, обусловленное избыточной продукцией гормона роста (ГР) и, как следствие, повышением уровня инсулиноподобного фактора роста 1 (ИФР-1).

Роль избыточной секреции ГР и ИФР-1 при COVID-19 в настоящее время неизвестна, однако данные факторы модулируют опосредованное гриппом А поражение легких у крыс [54]. Также известно, что синдром воспалительного цитокинового шторма, опосредованный ИЛ-6, о котором сообщают при COVID-19, подавляет ИФР-1 [55]. Хотя снижение ИФР-1 не было продемонстрировано, предполагалось, что это возможный фактор риска аутизма у младенцев от беременных матерей с COVID-19 [56]. Интересным перекрестным взаимодействием между системой ИФР-1 и рецептором АПФ2 для SARS-CoV-2 является общий вторичный мессенджер, включающий путь преобразователя сигнала и активатора транскрипции (STAT). Активация рецепторов АПФ2 цитокинами через энхансеры pan JAK-STAT была продемонстрирована в недавнем исследовании экспрессии мРНК [57]. Клиническая значимость этого неизвестна.

Избыточные уровни ГР и ИФР-1 оказывают патологическое воздействие на многие органы, приводя к широко распространенным метаболическим, респираторным и сердечно-сосудистым нарушениям. Респираторные осложнения возникают в первую очередь из-за отека мягких тканей и мышц носоглоточных ходов, гипертрофии хрящей и костей, сужения мелких дыхательных путей, расстройства регуляции дыхательных мышц и изменения архитектуры ребер и позвоночника, а также переломов костей [58]. Принимая во внимание наличие структурных изменений, можно предположить, что осложнения акромегалии могут усугублять тяжесть острых респираторных вирусных инфекций, включая COVID-19. Luty J. и соавт. описали случай тяжелой инфекции SARS-CoV-2 на фоне впервые диагностированной акромегалии [59]. Пациент поступил на 3-и сутки от начала заболевания с прогрессирующей лихорадкой, болью в верхней части живота, одышкой и периодическими синкопальными состояниями. В связи с наличием классической клинической картины (характерные черты лица, прогнатизм, большой нос и утолщенные губы, черный акантоз и акрохордоны), предиабета, недавно диагностированного синдрома обструктивного апноэ сна и повышения уровня ИФР-1 верифицирован диагноз акромегалии. Проведение МРТ гипофиза было отложено из-за подтвержденного у пациента COVID-19. После инициации терапии октреотидом отмечено значимое улучшение самочувствия, респираторного статуса и лабораторных показателей, что свидетельствует о том, что повышенные уровни ГР, ИФР-1 и иммуномодулирующие эффекты нелеченой акромегалии способствовали тяжелому течению COVID-19. Таким образом, акромегалия может быть ранее неизвестным фактором риска более тяжелого течения COVID-19. Нередко сопутствующие данному заболеванию гипокортицизм, сахарный диабет, гипертензия и переломы ухудшают прогноз больных. Для большинства пациентов сакромегалией основным методом лечения является трансназальная транссфеноидальная аденомэктомия, которая была запланирована для лечения пациента после карантина и выздоровления от COVID-19.

Однако поскольку акромегалия, наряду с другими синдромами, вызванными опухолями гипофиза, встречается относительно редко, реальных данных о ее лечении в эпоху COVID-19 недостаточно. В связи с этим в исследовании ACROCOVID был проведен международный онлайн-опрос о том, как изменились диагностика и лечение больных с акромегалией в новых условиях [60]. Отвечавшие на вопросы пациенты, эндокринологи, нейрохирурги и сестры, работающие в нейрохирургии, отметили, как и ожидалось, что наибольшее затруднение вызывала диагностика основного заболевания, а также осложнения, вызванные COVID-19 и их компенсация. 33,3% респондентов сообщили о задержке плановых хирургических вмешательств.

В пандемию из-за отсрочки оперативного лечения возросла роль медикаментозной терапии, особенно применение тех препаратов, которые больной может ввести себе сам, не обращаясь за помощью медицинского персонала. Безусловно, ланреотид и пасиреотид (off-label) имеют преимущества перед синтетическими аналогами октреотида пролонгированного действия, а нежелательный гипергликемический эффект пасиреотида может потенцироваться с негативным влиянием SARS-CoV-2 на β-клетки поджелудочной железы. Препарат выбора при сочетании COVID-19 и акромегалии — пэгвисомант. В перспективе обнадеживает факт появления перорального агониста соматостатина, что поможет пациентам с акромегалией еще качественнее контролировать заболевание [61].

Подытоживая вышесказанное, в период пандемии целесообразны отсрочка оперативного лечения и назначение медикаментозной терапии. Однако важно учитывать особенности течения основного заболевания у каждого пациента с акромегалией и выбирать тактику лечения индивидуально.

## ГИПОНАТРИЕМИЯ

Гипонатриемия (синдром неадекватной секреции антидиуретического гормона (СНСАДГ)) является наиболее распространенным нарушением электролитного баланса у госпитализированных пациентов с любой патологией, независимо от пандемии, и встречается (при учете случаев с уровнем натрия в сыворотке менее 135 ммоль/л) примерно у 30% пациентов с пневмонией.

Инфекционные заболевания и воспалительные состояния являются эффективными триггерами неосмотической индукции секреции вазопрессина. В этих ситуациях секреция вазопрессина в основном обусловлена ИЛ-6, который высвобождается моноцитами и макрофагами, преодолевает гематоэнцефалический барьер и активирует рецепторы ИЛ-6. Это, в свою очередь, вызывает высвобождение вазопрессина супраоптическим и паравентрикулярным ядрами гипоталамуса [62]. Другим механизмом является гиповолемическая гипонатриемия в результате диареи, рвоты и уменьшения перорального приема пищи. СНСАДГ и гиповолемия в равной степени были причиной гипонатриемии в ретроспективном многоцентровом обсервационном когортном исследовании у пациентов с COVID-19 в четырех больницах США [63].

Гипонатриемия наблюдалась у 34% включенных пациентов с COVID-19 (65 из 193) по сравнению с 24% пациентов с отрицательным результатом на SARS-CoV-2 (33 из 138). У 12% всех пациентов с COVID-19 (23 из 193) в ходе госпитализации наблюдались как гипонатриемия, так и гипернатриемия [64].

Результаты крупного международного реестра, в который включены 4664 госпитализированных пациента с COVID-19, исследования Health Outcome Predictive Evaluation for COVID-19 (HOPE) показали, что у 20,5% была гипонатриемия при поступлении. Тяжелая гипонатриемия (<120 ммоль/л) встречалась достаточно редко, всего у 0,4% пациентов. Позднее было описано исследование, включающее 16 пациентов, госпитализированных в стационар по поводу COVID-19, которое показало, что у 50% из них была гипонатриемия [65].

В нашем центре среди 153 пациентов, госпитализированных для стационарного лечения COVID-19 с мая по июнь 2020 г., медиана натриемии составила 137 ммоль/л, а гипонатриемия (Na<135 ммоль/л) выявлена у 25%, при этом тяжелая гипонатриемия (Na<130 ммоль/л) — у 4,6% пациентов [66].

Гипонатриемия является независимым предиктором тяжести COVID-19, включая смертность и потребность в ИВЛ [67]. Связь между содержанием натрия в плазме и смертностью от COVID-19 имеет U-образную форму. Ретроспективное исследование пациентов с подтвержденным SARS-CoV-2 в семи странах показало, что как гипонатриемия, так и гипернатриемия связаны с более высокой смертностью, но только гипонатриемия была связана с госпитализацией в отделение интенсивной терапии [63]. Вероятно, это было связано с тем, что пациенты с гипернатриемией были старше, имели больше сопутствующих заболеваний и, следовательно, реже попадали в ОРИТ ввиду более раннего летального исхода.

Пациентов с гипонатриемией и COVID-19 следует продолжать лечить в соответствии с общепринятыми рекомендациями [68]. Ограничение жидкости является основой лечения СНСАДГ, связанного с COVID-19. В серии случаев СНСАДГ, связанного сCOVID-19, ограничение жидкости от 750 мл до 1200 мл в день улучшало уровень натрия в плазме [69]. Как и у лиц без COVID-19, при лечении острой симптоматической гипонатриемии, проявляющейся судорогами и снижением уровня сознания, следует рассмотреть возможность инфузии гипертонического раствора. В проведенном исследовании при изучении COVID-19 в сочетании с СНСАДГ, проявляющимся острой тяжелой гипонатриемией, ограничение жидкости и инфузия гипертонического солевого раствора нормализовали уровень натрия в плазме к 4-му дню госпитализации [65]. Авторы предлагают проверять осмоляльность сыворотки и мочи, натрий в моче, тиреоидный профиль и утренний кортизол у пациентов с необъяснимой умеренной или тяжелой гипонатриемией.

Таким образом, гипонатриемия является предиктором тяжести COVID-19. Тщательный сбор анамнеза и оценка степени выраженности гипонатриемии важны для дифференциации между обезвоженным пациентом, которому требуется восполнение жидкости, и пациентом с эуволемией и СНСАДГ, которому требуется ограничение жидкости.

## ВАКЦИНАЦИЯ

Вакцинация против COVID-19 остается единственным эффективным методом борьбы с SARS-CoV-2. Доступность вакцинации должна быть гарантирована для всех, особенно для пациентов с высоким риском тяжелого течения COVID-19, которыми являются нейроэндокринные пациенты. Опубликованные в настоящее время данные не имеют информации о побочных эффектах вакцинации и о противопоказаниях к ней у пациентов с нейроэндокринными заболеваниями [70]. Несмотря на это, ряд авторов высказывают предположения о необходимости коррекции доз ГК перед вакцинацией у пациентов с надпочечниковой недостаточностью [71]. Будущие исследования в этой области крайне необходимы.

## ЗАКЛЮЧЕНИЕ

Пациенты с нейроэндокринными заболеваниями склонны к более тяжелому клиническому течению COVID-19 и значительному увеличению смертности. Следовательно, соблюдение мер профилактики заражения COVID-19 и вакцинация являются простой, но эффективной стратегией ведения подобных пациентов. Кроме того, пациенты должны быть осведомлены о возможности ухудшения течения основного заболевания и методах его коррекции, показаниях к экстренной госпитализации и пр. Необходимо расширить возможности телемедицины, включая междисциплинарное консультирование со специалистами смежных специальностей. Более того, такие стратегии, разработанные в условиях ограничений, вызванных пандемией, вполне могут в будущем стать новыми стандартами лечения нейроэндокринных заболеваний.

В настоящее время следует обратить особое внимание на систематический сбор данных о пациентах с нейроэндокринными заболеваниями и COVID-19 и использовать их для разработки инновационных стратегий ведения подобных пациентов.

## ДОПОЛНИТЕЛЬНАЯ ИНФОРМАЦИЯ

Источник финансирования. Работа выполнена по инициативе авторов без привлечения финансирования.

Конфликт интересов. Авторы декларируют отсутствие явных и потенциальных конфликтов интересов, связанных с публикацией настоящей статьи.

Участие авторов. Буйваленко У.В., Перепелова М.А., Золотарева Р.А. — анализ литературы, написание текста, подготовка рисунков; Белая Ж.Е., Мельниченко Г.А. — редактирование и финальное утверждение рукописи. Все авторы одобрили финальную версию статьи перед публикацией, выразили согласие нести ответственность за все аспекты работы, подразумевающую надлежащее изучение и решение вопросов, связанных с точностью или добросовестностью любой части работы.

## References

[cit1] Carvalho T, Krammer F, Iwasaki A. The first 12 months of COVID-19: a timeline of immunological insights. Nat Rev Immunol. 2021;21(4):245-256. doi: https://doi.org/10.1038/s41577-021-00522-1 33723416 PMC7958099

[cit2] Zhou Peng, Yang Xing-Lou, Wang Xian-Guang, Hu Ben, Zhang Lei, Zhang Wei, Si Hao-Rui, Zhu Yan, Li Bei, Huang Chao-Lin, Chen Hui-Dong, Chen Jing, Luo Yun, Guo Hua, Jiang Ren-Di, Liu Mei-Qin, Chen Ying, Shen Xu-Rui, Wang Xi, Zheng Xiao-Shuang, Zhao Kai, Chen Quan-Jiao, Deng Fei, Liu Lin-Lin, Yan Bing, Zhan Fa-Xian, Wang Yan-Yi, Xiao Geng-Fu, Shi Zheng-Li (2020). A pneumonia outbreak associated with a new coronavirus of probable bat origin. Nature.

[cit3] Chen Nanshan, Zhou Min, Dong Xuan, Qu Jieming, Gong Fengyun, Han Yang, Qiu Yang, Wang Jingli, Liu Ying, Wei Yuan, Xia Jia'an, Yu Ting, Zhang Xinxin, Zhang Li (2020). Epidemiological and clinical characteristics of 99 cases of 2019 novel coronavirus pneumonia in Wuhan, China: a descriptive study. The Lancet.

[cit4] Martins-Filho Paulo Ricardo, Tavares Carolina Santos Souza, Santos Victor Santana (2020). Factors associated with mortality in patients with COVID-19. A quantitative evidence synthesis of clinical and laboratory data. European Journal of Internal Medicine.

[cit5] Zhou Fei, Yu Ting, Du Ronghui, Fan Guohui, Liu Ying, Liu Zhibo, Xiang Jie, Wang Yeming, Song Bin, Gu Xiaoying, Guan Lulu, Wei Yuan, Li Hui, Wu Xudong, Xu Jiuyang, Tu Shengjin, Zhang Yi, Chen Hua, Cao Bin (2020). Clinical course and risk factors for mortality of adult inpatients with COVID-19 in Wuhan, China: a retrospective cohort study. The Lancet.

[cit6] (2020). Clinical and epidemiological characteristics of Coronavirus Disease 2019 (COVID-19) patients.

[cit7] Wu Yeshun, Xu Xiaolin, Chen Zijun, Duan Jiahao, Hashimoto Kenji, Yang Ling, Liu Cunming, Yang Chun (2020). Nervous system involvement after infection with COVID-19 and other coronaviruses. Brain, Behavior, and Immunity.

[cit8] Turnbull A.V., Rivier C. (2002). Regulation of the HPA Axis by Cytokines. Brain, Behavior, and Immunity.

[cit9] Leow Melvin Khee-Shing, Kwek Daniel Seow-Khee, Ng Alan Wei-Keong, Ong Kian-Chung, Kaw Gregory Jon-Leng, Lee Lawrence Soon-U (2005). Hypocortisolism in survivors of severe acute respiratory syndrome (SARS). Clinical Endocrinology.

[cit10] Alzahrani Ali S., Mukhtar Noha, Aljomaiah Abeer, Aljamei Hadeel, Bakhsh Abdulmohsen, Alsudani Nada, Elsayed Tarek, Alrashidi Nahlah, Fadel Roqayh, Alqahtani Eman, Raef Hussein, Butt Muhammad Imran, Sulaiman Othman (2020). The Impact of COVID-19 Viral Infection on the Hypothalamic-Pituitary-Adrenal Axis. Endocrine Practice.

[cit11] Solorio-Pineda Saúl, Almendárez-Sánchez César Adán, Tafur-Grandett Abrahan Alfonso, Ramos-Martínez Gabriel Arturo, Huato-Reyes Raúl, Ruiz-Flores Milton Inocencio, Sosa-Najera Antonio (2020). Pituitary macroadenoma apoplexy in a severe acute respiratory syndrome-coronavirus-2-positive testing: Causal or casual?. Surgical Neurology International.

[cit12] Taneja Charit, Fazeli Pouneh K., Gardner Paul A., Wang Eric W., Snyderman Carl H., Mahmud Hussain (2022). Rapidly Progressive Pituitary Apoplexy in a Patient with COVID-19 Disease Treated with Endoscopic Endonasal Surgery. Journal of Neurological Surgery Reports.

[cit13] Kamel Walaa A., Najibullah Mustafa, Saleh Mamdouh S., Azab Waleed A. (2021). Coronavirus disease 2019 infection and pituitary apoplexy: A causal relation or just a coincidence? A case report and review of the literature. Surgical Neurology International.

[cit14] Fleseriu Maria, Buchfelder Michael, Cetas Justin S., Fazeli Pouneh K., Mallea-Gil Susana M., Gurnell Mark, McCormack Ann, Pineyro Maria M., Syro Luis V., Tritos Nicholas A., Marcus Hani J. (2020). Pituitary society guidance: pituitary disease management and patient care recommendations during the COVID-19 pandemic—an international perspective. Pituitary.

[cit15] Chan Julie L., Gregory Kimberly D., Smithson Sarah S., Naqvi Mariam, Mamelak Adam N. (2020). Pituitary apoplexy associated with acute COVID-19 infection and pregnancy. Pituitary.

[cit16] Cain Derek W., Cidlowski John A. (2017). Immune regulation by glucocorticoids. Nature Reviews Immunology.

[cit17] Zhang Ning, Truong-Tran Quynh Ai, Tancowny Brian, Harris Kathleen E., Schleimer Robert P. (2014). Glucocorticoids Enhance or Spare Innate Immunity: Effects in Airway Epithelium Are Mediated by CCAAT/Enhancer Binding Proteins. The Journal of Immunology.

[cit18] Hasenmajer Valeria, Sbardella Emilia, Sciarra Francesca, Minnetti Marianna, Isidori Andrea M., Venneri Mary Anna (2020). The Immune System in Cushing’s Syndrome. Trends in Endocrinology & Metabolism.

[cit19] Martins-Filho Paulo Ricardo, Tavares Carolina Santos Souza, Santos Victor Santana (2020). Factors associated with mortality in patients with COVID-19. A quantitative evidence synthesis of clinical and laboratory data. European Journal of Internal Medicine.

[cit20] Fareau Gilbert G., Vassilopoulou-Sellin Rena (2007). Hypercortisolemia and Infection. Infectious Disease Clinics of North America.

[cit21] Baglin A, Hanslik T. Systemic corticosteroid treatment and risk of infectious diseases. Rev Med Interne. 2007;28(12):841-851. 17629359 10.1016/j.revmed.2007.05.030

[cit22] Lionakis Michail S, Kontoyiannis Dimitrios P (2003). Glucocorticoids and invasive fungal infections. The Lancet.

[cit23] Steffensen Charlotte, Bak Ann Mosegaard, Zøylner Rubeck Kristine, Jørgensen Jens Otto Lunde (2010). Epidemiology of Cushing’s Syndrome. Neuroendocrinology.

[cit24] Fardet L., Flahault A., Kettaneh A., Tiev K.P., Généreau T., Tolédano C., Lebbé C., Cabane J. (2007). Corticosteroid-induced clinical adverse events: frequency, risk factors and patient?s opinion. British Journal of Dermatology.

[cit25] Morgan Stuart A., McCabe Emma L., Gathercole Laura L., Hassan-Smith Zaki K., Larner Dean P., Bujalska Iwona J., Stewart Paul M., Tomlinson Jeremy W., Lavery Gareth G. (2014). 11β-HSD1 is the major regulator of the tissue-specific effects of circulating glucocorticoid excess. Proceedings of the National Academy of Sciences.

[cit26] Favalli Ennio Giulio, Bugatti Serena, Klersy Catherine, Biggioggero Martina, Rossi Silvia, De Lucia Orazio, Bobbio-Pallavicini Francesca, Murgo Antonella, Balduzzi Silvia, Caporali Roberto, Montecucco Carlomaurizio (2020). Impact of corticosteroids and immunosuppressive therapies on symptomatic SARS-CoV-2 infection in a large cohort of patients with chronic inflammatory arthritis. Arthritis Research & Therapy.

[cit27] Nissen Christoffer B, Sciascia Savino, de Andrade Danieli, Atsumi Tatsuya, Bruce Ian N, Cron Randy Q, Hendricks Oliver, Roccatello Dario, Stach Ksenija, Trunfio Mattia, Vinet Évelyne, Schreiber Karen (2021). The role of antirheumatics in patients with COVID-19. The Lancet Rheumatology.

[cit28] (2020). Dexamethasone in Hospitalized Patients with Covid-19. New England Journal of Medicine.

[cit29] Fardet L., Petersen I., Nazareth I. (2012). Risk of cardiovascular events in people prescribed glucocorticoids with iatrogenic Cushing's syndrome: cohort study. BMJ.

[cit30] Medhi Bikash, Sarma Phulen, Bhattacharyya Anusuya, Kaur Hardeep, Prajapat Manisha, Prakash Ajay, Kumar Subodh, Bansal Seema, Kirubakaran Richard, Reddy DibbantiHarikrishna, Muktesh Gaurav, Kaushal Karanvir, Sharma Saurabh, Shekhar Nishant, Avti Pramod, Thota Prasad (2021). Efficacy and safety of steroid therapy in COVID-19: A rapid systematic review and Meta-analysis. Indian Journal of Pharmacology.

[cit31] Peeters B., Boonen E., Langouche L., Van den Berghe G. (2014). The HPA axis response to critical illness: New study results with diagnostic and therapeutic implications. Molecular and Cellular Endocrinology.

[cit32] Edwards Christopher (2020). New Horizons: Does Mineralocorticoid Receptor Activation by Cortisol Cause ATP Release and COVID-19 Complications?. The Journal of Clinical Endocrinology & Metabolism.

[cit33] Funder John (2017). Aldosterone and Mineralocorticoid Receptors—Physiology and Pathophysiology. International Journal of Molecular Sciences.

[cit34] Karan Abhinav, Ali Kabeer, Rambaran Kirin, Del Corral Pedro, Sakhamuri Sateesh, Teelucksingh Surujpal (2020). COVID-19 and ethnicity: Does reduced responsiveness to glucocorticoids explain the more aggressive nature of disease among minorities?. Medical Hypotheses.

[cit35] CHARMANDARI EVANGELIA, KINO TOMOSHIGE, CHROUSOS GEORGE P. (2004). Familial/Sporadic Glucocorticoid Resistance: Clinical Phenotype and Molecular Mechanisms. Annals of the New York Academy of Sciences.

[cit36] Pivonello Rosario, Ferrigno Rosario, Isidori Andrea M, Biller Beverly M K, Grossman Ashley B, Colao Annamaria (2020). COVID-19 and Cushing's syndrome: recommendations for a special population with endogenous glucocorticoid excess. The Lancet Diabetes & Endocrinology.

[cit37] Yuen Kevin C.J., Blevins Lewis S., Findling James W. (2020). Important Management Considerations In Patients With Pituitary Disorders During The Time Of The Covid-19 Pandemic. Endocrine Practice.

[cit38] Pivonello Rosario, Isidori Andrea M, De Martino Maria Cristina, Newell-Price John, Biller Beverly M K, Colao Annamaria (2016). Complications of Cushing's syndrome: state of the art. The Lancet Diabetes & Endocrinology.

[cit39] Newell-Price John, Nieman Lynnette K, Reincke Martin, Tabarin Antoine (2020). ENDOCRINOLOGY IN THE TIME OF COVID-19: Management of Cushing’s syndrome. European Journal of Endocrinology.

[cit40] Guarnotta Valentina, Ferrigno Rosario, Martino Marianna, Barbot Mattia, Isidori Andrea M., Scaroni Carla, Ferrante Angelo, Arnaldi Giorgio, Pivonello Rosario, Giordano Carla (2020). Glucocorticoid excess and COVID-19 disease. Reviews in Endocrine and Metabolic Disorders.

[cit41] Levi Marcel, Thachil Jecko, Iba Toshiaki, Levy Jerrold H (2020). Coagulation abnormalities and thrombosis in patients with COVID-19. The Lancet Haematology.

[cit42] Wagner Jeffrey, Langlois Fabienne, Lim Dawn Shao Ting, McCartney Shirley, Fleseriu Maria (2019). Hypercoagulability and Risk of Venous Thromboembolic Events in Endogenous Cushing's Syndrome: A Systematic Meta-Analysis. Frontiers in Endocrinology.

[cit43] St-Jean Matthieu, Lim Dawn S.T., Langlois Fabienne (2021). Hypercoagulability in Cushing’s syndrome: From arterial to venous disease. Best Practice & Research Clinical Endocrinology & Metabolism.

[cit44] Yuno Akiko, Kenmotsu Yoshiyuki, Takahashi Yuka, Nomoto Hiroshi, Kameda Hiraku, Cho Kyu Yong, Nakamura Akinobu, Yamashita Yu, Nakamura Junichi, Nakakubo Sho, Kamada Keisuke, Suzuki Masaru, Sugino Hirokazu, Inoshita Naoko, Konno Satoshi, Miyoshi Hideaki, Atsumi Tatsuya, Sawamura Yutaka, Shimatsu Akira (2020). Successful management of a patient with active Cushing’s disease complicated with coronavirus disease 2019 (COVID-19) pneumonia. Endocrine Journal.

[cit45] Kakodkar Pramath, Kaka Nagham, Baig MN (2020). A Comprehensive Literature Review on the Clinical Presentation, and Management of the Pandemic Coronavirus Disease 2019 (COVID-19). Cureus.

[cit46] Vaduganathan Muthiah, Vardeny Orly, Michel Thomas, McMurray John J.V., Pfeffer Marc A., Solomon Scott D. (2020). Renin–Angiotensin–Aldosterone System Inhibitors in Patients with Covid-19. New England Journal of Medicine.

[cit47] Qiao Yao, Shin Jung-Im, Chen Teresa K., Inker Lesley A., Coresh Josef, Alexander G. Caleb, Jackson John W., Chang Alex R., Grams Morgan E. (2020). Association Between Renin-Angiotensin System Blockade Discontinuation and All-Cause Mortality Among Persons With Low Estimated Glomerular Filtration Rate. JAMA Internal Medicine.

[cit48] European Society of Cardiology. Position statement of the ESC council on hypertension on ACE-inhibitors and angio- tensin receptor blockers [Internet]. Sophia Antipolis: ESC; 2020 [cited 2020 Apr 20]. Available from: https://www.es-cardio.org/Councils/Council-on-Hypertension-(CHT)/News/position-statement-of-the-esc-council-on-hyperten-sion-on-ace-inhibitors-and-ang

[cit49] Formenti Anna Maria, Pedone Erika, di Filippo Luigi, Ulivieri Fabio Massimo, Giustina Andrea (2020). Are women with osteoporosis treated with denosumab at risk of severe COVID-19?. Endocrine.

[cit50] Belaya Zhanna, Golounina Olga, Melnichenko Galina, Tarbaeva Natalia, Pashkova Evgenia, Gorokhov Maxim, Kalashnikov Viktor, Dzeranova Larisa, Fadeev Valentin, Volchkov Pavel, Dedov Ivan (2021). Clinical course and outcome of patients with ACTH-dependent Cushing’s syndrome infected with novel coronavirus disease-19 (COVID-19): case presentations. Endocrine.

[cit51] Webb Brandon J, Peltan Ithan D, Jensen Paul, Hoda Daanish, Hunter Bradley, Silver Aaron, Starr Nathan, Buckel Whitney, Grisel Nancy, Hummel Erika, Snow Gregory, Morris Dave, Stenehjem Eddie, Srivastava Rajendu, Brown Samuel M (2020). Clinical criteria for COVID-19-associated hyperinflammatory syndrome: a cohort study. The Lancet Rheumatology.

[cit52] de Filette J M K, Sol Bastiaan, Awada Gil, Andreescu Corina E, Unuane David, Aspeslagh Sandrine, Poelaert Jan, Bravenboer Bert (2022). COVID-19 and Cushing’s disease in a patient with ACTH-secreting pituitary carcinoma. Endocrinology, Diabetes & Metabolism Case Reports.

[cit53] Beretta Federica, Dassie Francesca, Parolin Matteo, Boscari Federico, Barbot Mattia, Busetto Luca, Mioni Roberto, De Carlo Eugenio, Scaroni Carla, Fallo Francesco, Vettor Roberto, Maffei Pietro (2020). Practical Considerations for the Management of Cushing's Disease and COVID-19: A Case Report. Frontiers in Endocrinology.

[cit54] Li Guiping, Zhou Lijuan, Zhang Can, Shi Yun, Dong Derong, Bai Miao, Wang Rong, Zhang Chuanfu (2019). Insulin-Like Growth Factor 1 Regulates Acute Inflammatory Lung Injury Mediated by Influenza Virus Infection. Frontiers in Microbiology.

[cit55] Ye Qing, Wang Bili, Mao Jianhua (2020). The pathogenesis and treatment of the `Cytokine Storm' in COVID-19. Journal of Infection.

[cit56] Steinman Gary (2020). COVID-19 and autism. Medical Hypotheses.

[cit57] Hennighausen Lothar, Lee Hye Kyung (2020). Activation of the SARS-CoV-2 Receptor <i>Ace2</i> by Cytokines Through Pan JAK-STAT Enhancers. SSRN Electronic Journal.

[cit58] Rees P. J., Hay J. G., Webb J. R. (2008). Acute exacerbation of upper airway obstruction in acromegaly. Postgraduate Medical Journal.

[cit59] Luty Jacob, Hayward LesleAnn, Jackson Melanie, Duell P Barton (2021). Severe respiratory failure in a patient with COVID-19 and acromegaly: rapid improvement after adding octreotide. BMJ Case Reports.

[cit60] Giustina Andrea, Legg Ewen, Cesana Bruno Mario, Frara Stefano, Mortini Pietro, Fleseriu Maria (2021). Results from ACROCOVID: an international survey on the care of acromegaly during the COVID-19 era. Endocrine.

[cit61] Lodish Maya B (2021). How do clinicians integrate oral octreotide into their treatment plan for acromegaly?. The Lancet Diabetes & Endocrinology.

[cit62] Swart Reinout M., Hoorn Ewout J., Betjes Michiel G., Zietse Robert (2010). Hyponatremia and Inflammation: The Emerging Role of Interleukin-6 in Osmoregulation. Nephron Physiology.

[cit63] Frontera Jennifer A., Valdes Eduard, Huang Joshua, Lewis Ariane, Lord Aaron S., Zhou Ting, Kahn D. Ethan, Melmed Kara, Czeisler Barry M., Yaghi Shadi, Scher Erica, Wisniewski Thomas, Balcer Laura, Hammer Elizabeth (2020). Prevalence and Impact of Hyponatremia in Patients With Coronavirus Disease 2019 in New York City. Critical Care Medicine.

[cit64] Voets Philip JGM, Frölke Sophie C, Vogtländer Nils PJ, Kaasjager Karin AH (2021). COVID-19 and dysnatremia: A comparison between COVID-19 and non-COVID-19 respiratory illness. SAGE Open Medicine.

[cit65] Ho Kam Sing, Narasimhan Bharat, Kumar Aravinth, Flynn Erin, Salonia James, El-Hachem Karim, Mathew Joseph P. (2020). Syndrome of inappropriate antidiuretic hormone as the initial presentation of COVID-19: A novel case report. Nefrología.

[cit66] Abilov Zaur, Kononykhina Anastasia, Sardaeva Daria, Katamadze Nino, Pigarova Ekaterina, Dzeranova Larisa, Marova Evgenia, Olga Yu. Rebrova, Melnichenko Galina, Mokrysheva Natalia (2021). Electrolyte disturbances in hospitalized patients with COVID-19. Endocrine Abstracts.

[cit67] Ruiz-Sánchez Jorge Gabriel, Núñez-Gil Ivan J., Cuesta Martin, Rubio Miguel A., Maroun-Eid Charbel, Arroyo-Espliguero Ramón, Romero Rodolfo, Becerra-Muñoz Victor Manuel, Uribarri Aitor, Feltes Gisela, Trabattoni Daniela, Molina María, García Aguado Marcos, Pepe Martino, Cerrato Enrico, Alfonso Emilio, Castro Mejía Alex Fernando, Roubin Sergio Raposeiras, Buzón Luis, Bondia Elvira, Marin Francisco, López Pais Javier, Abumayyaleh Mohammad, D’Ascenzo Fabrizio, Rondano Elisa, Huang Jia, Fernandez-Perez Cristina, Macaya Carlos, de Miguel Novoa Paz, Calle-Pascual Alfonso L., Estrada Perez Vicente, Runkle Isabelle (2020). Prognostic Impact of Hyponatremia and Hypernatremia in COVID-19 Pneumonia. A HOPE-COVID-19 (Health Outcome Predictive Evaluation for COVID-19) Registry Analysis. Frontiers in Endocrinology.

[cit68] Christ-Crain Mirjam, Hoorn Ewout J, Sherlock Mark, Thompson Chris J, Wass John A H (2020). ENDOCRINOLOGY IN THE TIME OF COVID-19: Management of diabetes insipidus and hyponatraemia. European Journal of Endocrinology.

[cit69] Yousaf Zohaib, Al-Shokri Shaikha D., Al-soub Hussam, Mohamed Mouhand F. H. (2020). COVID-19-associated SIADH: a clue in the times of pandemic!. American Journal of Physiology-Endocrinology and Metabolism.

[cit70] Polack Fernando P., Thomas Stephen J., Kitchin Nicholas, Absalon Judith, Gurtman Alejandra, Lockhart Stephen, Perez John L., Pérez Marc Gonzalo, Moreira Edson D., Zerbini Cristiano, Bailey Ruth, Swanson Kena A., Roychoudhury Satrajit, Koury Kenneth, Li Ping, Kalina Warren V., Cooper David, Frenck Robert W., Hammitt Laura L., Türeci Özlem, Nell Haylene, Schaefer Axel, Ünal Serhat, Tresnan Dina B., Mather Susan, Dormitzer Philip R., Şahin Uğur, Jansen Kathrin U., Gruber William C. (2020). Safety and Efficacy of the BNT162b2 mRNA Covid-19 Vaccine. New England Journal of Medicine.

[cit71] Baden Lindsey R., El Sahly Hana M., Essink Brandon, Kotloff Karen, Frey Sharon, Novak Rick, Diemert David, Spector Stephen A., Rouphael Nadine, Creech C. Buddy, McGettigan John, Khetan Shishir, Segall Nathan, Solis Joel, Brosz Adam, Fierro Carlos, Schwartz Howard, Neuzil Kathleen, Corey Lawrence, Gilbert Peter, Janes Holly, Follmann Dean, Marovich Mary, Mascola John, Polakowski Laura, Ledgerwood Julie, Graham Barney S., Bennett Hamilton, Pajon Rolando, Knightly Conor, Leav Brett, Deng Weiping, Zhou Honghong, Han Shu, Ivarsson Melanie, Miller Jacqueline, Zaks Tal (2020). Efficacy and Safety of the mRNA-1273 SARS-CoV-2 Vaccine. New England Journal of Medicine.

